# A Case of Inherited t(4;10)(q26;q26.2) Chromosomal Translocation Elucidated by Multiple Chromosomal and Molecular Analyses. Case Report and Review of the Literature

**DOI:** 10.3390/genes12121957

**Published:** 2021-12-07

**Authors:** Roxana Popescu, Mihaela Grămescu, Lavinia Caba, Monica-Cristina Pânzaru, Lăcrămioara Butnariu, Elena Braha, Setalia Popa, Cristina Rusu, Georgeta Cardos, Monica Zeleniuc, Violeta Martiniuc, Cristina Gug, Luminiţa Păduraru, Maria Stamatin, Carmen C. Diaconu, Eusebiu Vlad Gorduza

**Affiliations:** 1 Medical Genetics Department, “Grigore T. Popa” University of Medicine and Pharmacy, 16 University Street, 700115 Iasi, Romania; roxana.popescu2014@gmail.com (R.P.); mihaelagramescu@yahoo.ro (M.G.); monica.panzaru@yahoo.com (M.-C.P.); lacrybutnariu@yahoo.com (L.B.); setalia_ostapov@yahoo.com (S.P.); abcrusu@gmail.com (C.R.); vgord@mail.com (E.V.G.); 2 “C. I. Parhon” National Institute of Endocrinology, 34-35 Aviatorilor Avenue, 011853 Bucharest, Romania; elenabraha@yahoo.com; 3 Personal Genetics Laboratory Bucharest, 4 Strada Frumoasa Street, 010987 Bucharest, Romania; cardosg@yahoo.com (G.C.); monistoian_dr@yahoo.com (M.Z.); 4 Medical Genetics Department, “Carol Davila” University of Medicine and Pharmacy, 8 Eroii Sanitari Avenue, 050474 Bucharest, Romania; 5 Medical Genetics Department, “Cuza-Vodă” Obstetrics and Gynecology Hospital, 34 Cuza Voda Street, 700038 Iasi, Romania; vyo_genetics@yahoo.com; 6 Microscopic Morphology Department, “Victor Babes” University of Medicine and Pharmacy, 2 Piata Eftimie Murgu, 300041 Timișoara, Romania; 7 Neonatology Department, “Grigore T. Popa” University of Medicine and Pharmacy, 16 University Street, 700115 Iasi, Romania; luminita.paduraru@gmail.com (L.P.); maria.stamatin@yahoo.com (M.S.); 8 Stefan S. Nicolau Institute of Virology, Romanian Academy, 285 Mihai Bravu, 030304 Bucharest, Romania; ccdiaconu@yahoo.com

**Keywords:** 4q partial trisomy, 10q microdeletion, 4q35.2 triplication, multiple congenital anomalies syndrome, cytogenetic analyses

## Abstract

We present a complex chromosomal anomaly identified using cytogenetic and molecular methods. The child was diagnosed during the neonatal period with a multiple congenital anomalies syndrome characterized by: flattened occipital region; slight turricephaly; tall and broad forehead; hypertelorism; deep-set eyes; down slanting and short palpebral fissures; epicanthic folds; prominent nose with wide root and bulbous tip; microstomia; micro-retrognathia, large, short philtrum with prominent reliefs; low set, prominent ears; and congenital heart disease. The GTG banding karyotype showed a 46,XY,der(10)(10pter→10q26.2::4q26→4qter) chromosomal formula and his mother presented an apparently balanced reciprocal translocation: 46,XX,t(4;10)(q26;q26.2). The chromosomal anomalies of the child were confirmed by MLPA, and supplementary investigation discovered a quadruplication of the 4q35.2 region. The mother has a triplication of the same chromosomal fragment (4q35.2). Using array-CGH, we described the anomalies completely. Thus, the boy has a 71,057 kb triplication of the 4q26–q35.2 region, a 562 kb microdeletion in the 10q26.3 region, and a 795 kb quadruplication of the 4q35.2 region, while the mother presents a 795 kb triplication of the 4q35.2 region. Analyzing these data, we consider that the boy’s phenotype is influenced only by the 4q partial trisomy. We compare our case with similar cases, and we review the literature data.

## 1. Introduction

Congenital anomalies are structural defects produced during the prenatal period and are present at birth [1]. Usually, the severe syndromes with congenital anomalies (SCAs) are generated by an unbalanced chromosomal rearrangement that disrupts large numbers of developmentally important genes, which results in specific and complex phenotypes. The confirmation of a chromosomal anomaly requires GTG-banded chromosomal analysis in association with supplementary analyses, such as FISH, MLPA, or array-CGH [2,3]. 

Partial trisomy 4q is a rare unbalanced structural chromosomal anomaly that was first described in the early 1970s [4,5,6]. Since then, less than 100 cases have been described. Some anomalies were de novo, but the majority are derived from the malsegregation of a parental balanced chromosomal anomaly. In such cases, the phenotype is induced by the association of 4q partial trisomy with partial monosomy of a different chromosome [7]. The delineation of genotype–phenotype correlation in 4q trisomy is difficult because the described cases had different breakpoints and presented different associated monosomies and the ages of reported patients were variable [8,9].

Partial 10q monosomy was first described by Lewandowski et al. (1978), and since then, about 110 cases have been reported in the literature [10]. The chromosomal anomalies are: terminal 10q monosomy, terminal 10q monosomy associated with a partial trisomy of a different chromosome, and 10q26.1 microdeletion. The symptomatology is variable and depends on the type of anomaly, the fragment size, and coexistence with other anomalies. The phenotypic spectrum is heterogeneous, and it is characterized by some common clinical features, such as craniofacial anomalies, developmental delay, intellectual disability, urinary tract abnormalities, and heart defects [11,12,13,14,15,16].

We present a case with a multiple congenital anomalies syndrome, identified during the neonatal period, that has an association between partial 4q trisomy and partial 10q monosomy. These anomalies were generated by the malsegregation of the maternal chromosomes 4 and 10, which are involved in a balanced reciprocal translocation. The 4q fragment located on the derivative chromosome 10 also presents a small 4q35.2 duplication, also inherited from the mother.

## 2. Materials and Methods

### 2.1. Clinical Evaluation

The patient underwent a multidisciplinary evaluation, including the following specialties: genetics, pediatrics, cardiology, and neonatology.

The patient’s parents gave written informed consent (including for publication of images) considering the Declaration of Helsinki. This study was approved by the Ethics Committee for Scientific Research of the “Grigore T. Popa” University of Medicine and Pharmacy, Iasi, Romania.

### 2.2. Cytogenetic Analyses 

Peripheral blood lymphocytes were cultured in a growth medium (RPMI 1640, Gibco, Thermo Fisher Scientific Inc, Waltham, MA, USA). Metaphase chromosomes were harvested, and slides were made for analysis. GTG banding was used for staining (at the 550-band level). Chromosomal analysis was performed using CytoVision software 2.81 (Applied Imaging, Grand Rapids, MI, USA), and the aberrations and karyotypes were classified according to the International System for Human Cytogenomic Nomenclature (ISCN 2016).

### 2.3. MLPA Technique

We used commercially available P036 and P070 SALSA MLPA kits (MRC-Holland, Amsterdam, The Netherlands). These kits screen for subtelomeric copy number variations and contain one MLPA probe for each subtelomeric region except for the short arms of acrocentric chromosomes (13p, 14p, 15p, 21p, and 22p), for which a probe on the q arm, close to the centromere, is included instead. To confirm the abnormalities detected, we applied kits P264 (for subtelomeric regions of chromosomes 1q, 2q, 3q, and 4q) and P286 (for subtelomeric regions of chromosomes 9q, 10q, 11q, and 12q) (MRC-Holland, Amsterdam, The Netherlands).

The DNA extraction from peripheral blood was performed using the Wizard Genomic DNA Purification Kit (Promega Corp., Madison, WI, USA). The standard MLPA analysis was performed according to the manufacturer’s instructions. Briefly, 300 nanograms of genomic DNA were denatured and hybridized with SALSA probes at 60 °C for 16 h. After 15 min ligation at 54 °C, PCR was performed in a Gradient Palm-Cycler (Corbett Research, Mortlake, NSW, Australia), available in a 96-well format, using Cy5 universally labeled primers. Fluorescent amplification products were subsequently separated through capillary electrophoresis in a CEQ 8000 GeXP Genetic Analysis System (Beckman Coulter, Brea, CA, USA) sequencer and were analyzed using the default software. The number of DNA copies was estimated using the Coffalyser.NET V9 software (MRC-Holland, Amsterdam, The Netherlands), which calculates the ratio of peak areas in test samples over those of normal controls for each target sequence. Reference DNA samples were collected from healthy individuals (without developmental delay, intellectual disabilities, or congenital abnormalities), prepared using the same DNA extraction method, and previously evaluated for copy number variation of target areas. There was a minimum of 3 reference samples per run, randomly distributed, with one more for every 7 test samples.

### 2.4. Array-CGH Technique

Array Comparative Genomic Hybridization (aCGH) analysis was performed using the Sure Print G3 ISCA V2 CGH 8x60K Array Kit (Agilent Technologies®, Santa Clara, CA, USA), the NimbleGen MS 200 Microarray Scanner (Roche Applied Science, Basel, Switzerland), and NimbleGen MS 200 Software v1.1.

## 3. Results

### Clinical Presentation

**Figure 1:**
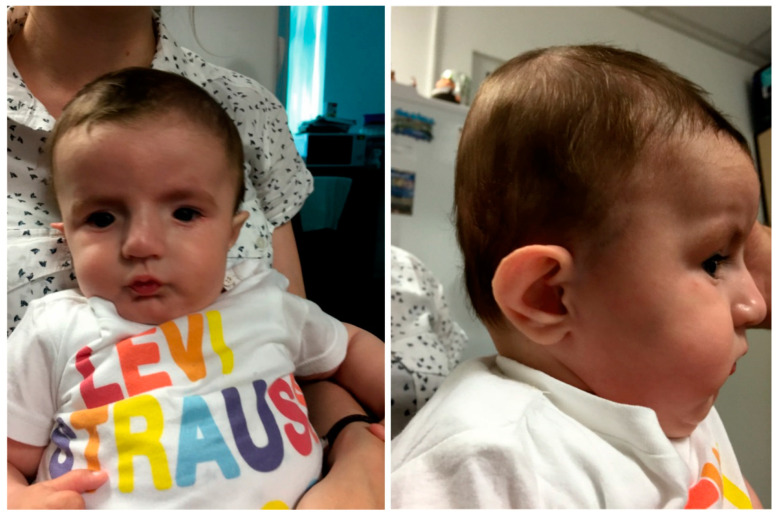
Cranio-facial aspect of the index case.

We present a boy with a mild development delay and multiple congenital anomalies syndrome, the result of a pregnancy supervised clinically and by prenatal screening. Prenatal ultrasound scan (second trimester) was normal and biochemical screening (maternal serum markers—alpha-fetoprotein, human chorionic gonadotropin, and unconjugated estriol at 16th weeks of pregnancy) showed values without risk for chromosomal abnormalities. Parents are both healthy, young (father—32 years, mother—28 years), unrelated, with no “known” family history of genetic disorders or birth defects. The child is the first offspring of the couple, and he was born at 38 WA, with a weight of 2350 gr, height 50 cm, head circumference 35 cm, and an Apgar score of 8 at 1 min. and 8 at 5 min. The patient was first examined during the neonatal period and was re-evaluated at 6 months. He presented some facial dysmorphic features: flattened skull in the occipital region, with slight turricephaly; tall and broad forehead; hypertelorism—inner canthal distance 2.7 cm (+2SD), outer canthal distance 6.3 cm (+2SD); interpupillary distance 4.6 cm (+2SD); deep-set eyes, down slanting and short palpebral fissures; epicanthic folds; prominent nose, with wide root and bulbous tip; microstomia and micro-retrognathia; large, short philtrum with prominent reliefs,; low set, prominent ears, with big scaphoid fossa and big cavum concha (Figure 1). The following skills were developed: follows moving objects with eyes; listens to and follows simple directions; cries, babbles, and coos; reaches for objects and grasps; puts objects in mouth; lifts head and sits with back straight; smiles spontaneously. The ultrasound scan revealed a persistent ductus arteriosus (ΔP = 5 mm, velocity = 1 m/s, with left-to-right shunting) and atrial septal defect ostium secundum (3 mm, with left-to-right shunting), but without changes in kidneys. The cardiologist decided that no surgery was needed.

**Figure 2:**
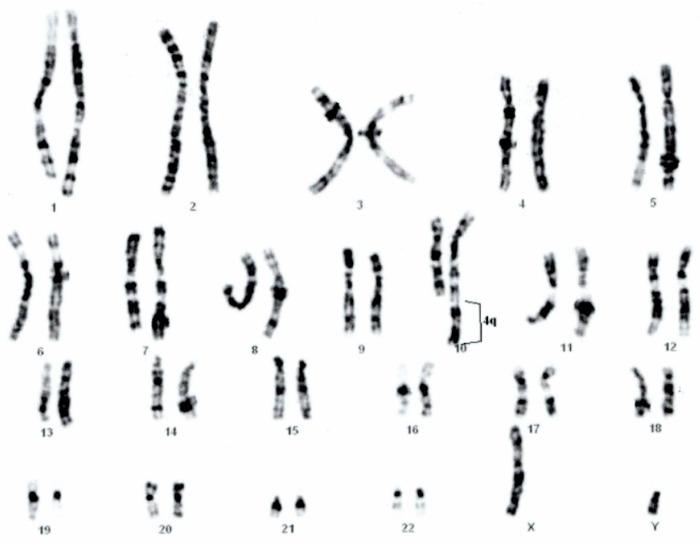
Child’s karyotype 46,XY,der(10)(10pter→10q26.2::4q26→4qter).

**Figure 3:**
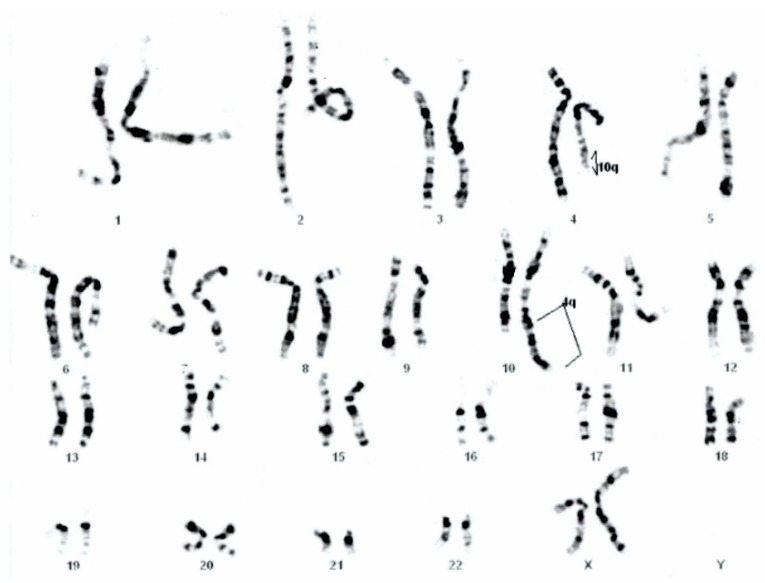
Mother’s karyotype 46,XX,t(4; 10)(q26; q26.2).

GTG banding showed a 46,XY,der(10)(10pter→10q26.2::4q26→4qter) chromosomal formula (Figure 2) that imposed parental karyotype. The father was normal, but the mother have a balanced reciprocal translocation between chromosomes 4 and 10: 46,XX,t(4;10)(q26;q26.2) (Figure 3).

Using the MLPA technique with telomere probes (P036 and P070, MRC-Holland, Amsterdam, The Netherlands), we discovered a 1.5 fold amplification of the 4q35.2-4q35.1 segment (concordant with a 4q partial trisomy) and a 0.5 fold amplification of the 10qter segment (concordant with a partial 10q monosomy); we also discovered a 2-fold amplification of the 4q35.2 segment (concordant with a 4q partial tetrasomy—a segment that includes the *ZPF42* and *TRIML2* genes). To validate these changes, we used MLPA probe P264 (for subtelomeric regions of chromosomes 1q, 2q, 3q, and 4q) and probe P286 (for subtelomeric regions of chromosomes 9q, 10q, 11q, and 12q).

**Figure 4:**
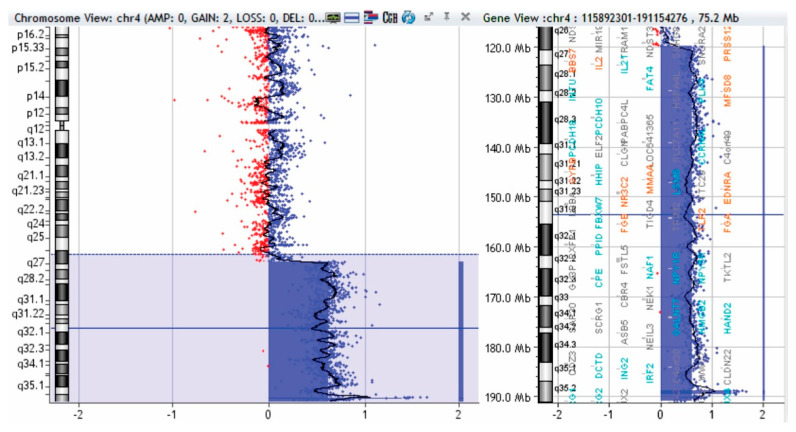
Array-CGH test of patient—triplication of 71,057 kb in the 4q26-q35.2 region (shaded in blue). Red and blue dots represent the log2 ratio of fluorescence (Cy5/Cy3), calculated by analytical software; log2 ratio at 1 indicates a duplication of the DNA of the region (3 copies) in the test sample versus the control (in blue color), and a log2 ratio at -1 indicates a deletion of the DNA of that region in the test sample versus the control (red deviation).

**Figure 5:**
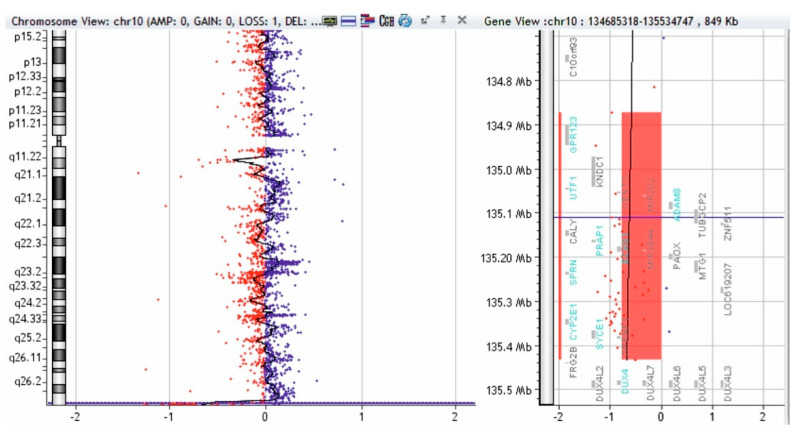
Array-CGH test of patient—microdeletion of 562 kb in the 10q26.3 region (shaded in red). Red and blue dots represent the log2 ratio of fluorescence (Cy5/Cy3), calculated by analytical software; log2 ratio at 1 indicates a duplication of the DNA of the region (3 copies) in the test sample versus the control (in blue color) and a log2 ratio at -1 indicates a deletion of the DNA of that region in the test sample versus the control (red deviation).

**Figure 6:**
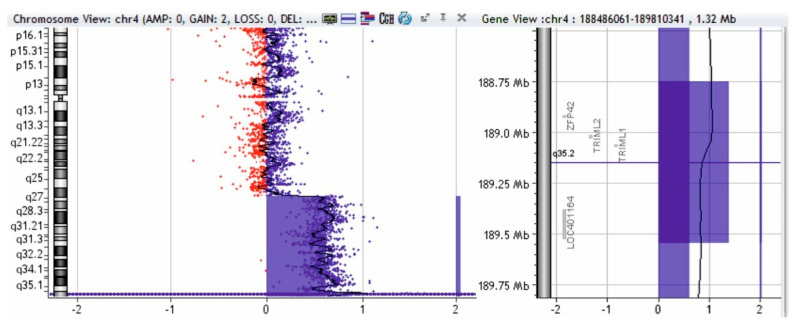
Array-CGH test of patient—quadruplication of 795 kb in the 4q35.2 region (shaded in blue). Red and blue dots represent the log2 ratio of fluorescence (Cy5/Cy3), calculated by analytical software; log2 ratio at 1 indicates a duplication of the DNA of the region (3 copies) in the test sample versus the control (in blue color), and a log2 ratio at -1 indicates a deletion of the DNA of that region in the test sample versus the control (red deviation).

To complete the genetic investigation, we applied an array-CGH that confirmed a 71,057 kb triplication of the 4q26-q35.2 region (genomic coordinates on chromosome 4: 119839900—190896674) (Figure 4), a 562 kb microdeletion of the 10q26.3 region (genomic coordinates on chromosome 10: 134872533—135434178) (Figure 5), and a 795 kb quadruplication of the 4q35.2 region (Figure 6).

**Figure 7:**
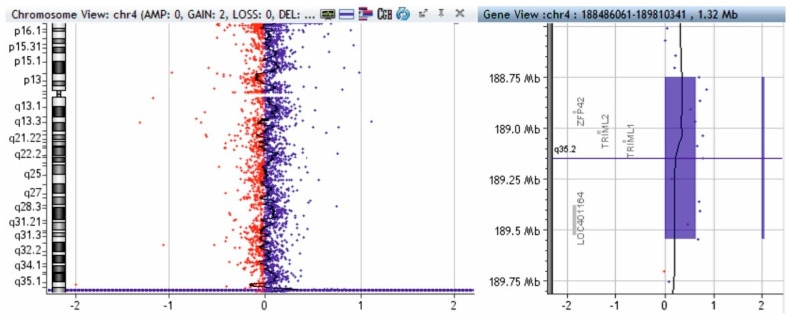
Array-CGH test of patient’s mother—triplication of 795 kb in the 4q35.2 region (shaded in blue). Red and blue dots represent the log2 ratio of fluorescence (Cy5/Cy3), calculated by analytical software; log2 ratio at 1 indicates a duplication of the DNA of the region (3 copies) in the test sample versus the control (in blue color) and a log2 ratio at -1 indicates a deletion of the DNA of that region in the test sample versus the control (red deviation).

The triplication of 71,057 kb of the 4q26–q35.2 region was pathogenic. The microdeletion of 562 kb in the 10q26.3 region is probably not pathogenic. To verify if the 4q35.2 quadruplication is pathogenic, we performed array-CGH on both parents, and the mother presented a triplication of 795 kb in the 4q35.2 region (Figure 7).

## 4. Discussion

We present a case that associates an important 4q partial trisomy (4q26–q35.2) and a small 10q partial monosomy (10q26.3). These chromosomal anomalies originate from the mother, who carries a balanced reciprocal translocation t(4q;10q)(q26;q26.3). Moreover, the son presents a 795 kb quadruplication in the 4q35.2 region, inherited from the maternal line. Due to the small size of the 10q partial monosomy, we could presume that the phenotype of our patient is determined mainly by the 4q partial trisomy.

Our case is the second cited in the literature. Zhang et al. (2009) presented a patient that associated a 4q partial trisomy (4q26–q35.2) and a 10q microdeletion (10q26.3) [7]. The breakpoints on chromosomes 4 and 10 are similar in these two cases. The breakpoints on chromosome 4q are located at 119,839,900 bp from the 4p telomere in our case and at 118,785,802 bp from the 4p telomere in the case presented by Zhang et al. (2009). The breakpoints on chromosome 10q are located at 134,872,533 bp from the 10p telomere in our case and at 134,750,859 bp from the 10 p telomere in the case presented by Zhang et al. (2009). Thus, in both cases, the size of the 4q duplication is approximately 71 Mb, while the 10q microdeletion’s size is approximately 0.55 Mb.

**Figure 8:**
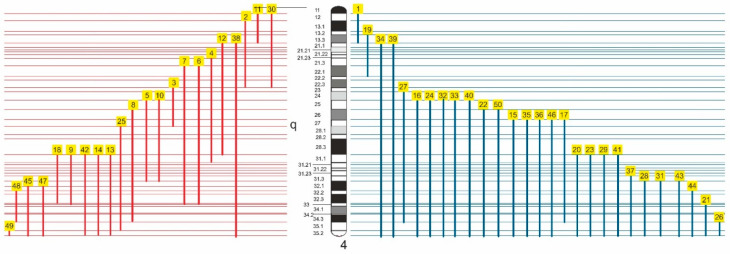
Ideogram of chromosome 4 and distribution of breakpoints in 4q partial trisomy. Red lines represent different 4q duplications identified by different authors; blue lines represent the cases with a translocation between chromosome 4 and other chromosomes; the numbers 1–50 represent the references presented in Table 1.

Trisomy 4q syndrome is a heterogeneous disorder because the reported patients have different breakpoints located on the long arm of chromosome 4; some patients associate different monosomies, and the age of reported patients vary between newborn and adulthood [17]. The breakpoints in trisomy 4q syndrome are distributed throughout the long arm of chromosome 4, except for the 4q11 band. The distribution of different types of 4q partial trisomy is presented in Figure 8.

Trisomy 4q syndrome shows a complex phenotype that includes growth retardation, intellectual disability, a specific craniofacial dysmorphism (microcephaly, epicanthic folds, high nasal bridge, short philtrum, microretrognathia, and low set and malformed ears), congenital heart defects (atrial or ventricular septal defect and patent ductus arteriosus), renal defects, and/or thumb anomalies (Table 2).

Celle et al. (2000) analyzed the duplications of the long arm of chromosome 4 and concluded that 4q partial trisomy could be generated by multiple genetic mechanisms, such as unbalanced translocation (the result of malsegregation of parental derivative chromosomes involved in a balanced reciprocal translocation) de novo duplication, de novo unbalanced translocation, and, more rarely, a chromosomal insertion [17]. Table 1 illustrates the heterogeneity of chromosomal anomalies in 4q partial trisomy. Thus, in 22 cases, the anomaly was de novo (16 cases with de novo duplication and 6 cases with de novo translocation). The other cases were inherited anomalies/unknown status of inheritance, and most of them (28 cases) are represented by different translocations between chromosome 4 and other chromosomes. Out of the translocations (de novo or inherited), the most frequent are: t(4;18) and t(4;21)—4 cases; t(4;13), t (2;4) and t(4;10)—3 cases; and t(4;8), t(4;9), and t(4;20), each of them with two cases.

Figure 8 shows the heterogeneity of the breakpoints’ position on the long arm of chromosome 4. Thus, we can divide 4q partial trisomy into four categories: proximal (between 4q12 and 4q23), intermediate (between 4q22–23 and 4q31), terminal (between 4q31 and 4qter), and large 4q partial trisomy (between 4q21–25 and 4qter). Phenotypic changes generated by these anomalies are different, and we will analyze them separately.

The proximal 4q partial trisomies are represented by the cases 1 [18], 2 [19], 11 [28], 19 [35], and 30 [43] from Table 1 and Figure 8. In all these cases, the phenotype is milder and the main characteristics are growth and psychomotor retardation, microcephaly, and low set malformed ears. For example, Shashi et al. (1999) reported a boy with a 4q12–q13 duplication that presented microcephaly and intellectual disability as main features [28]. Additionally, minimal phenotypic changes were reported by Bonnet et al. (2006), who presented a child with a 4q11–4q13.2 partial trisomy that had only a mild psychomotor delay [61]. There were similar findings by Matoso et al. (2013), who presented two siblings that have a 4q13.1–q13.3 partial trisomy that presented only delayed developmental milestones and attention-deficit hyperactivity disorder [62].

Additionally, a mild phenotype seems to be associated with a 4q partial trisomy of the 4q23–q27 region. Thus, Case 3 from Table 2 and Figure 8, reported by Halal et al. (1991), presented growth and psychomotor delay and nonspecific dysmorphic features such as epicanthic folds and a high nasal bridge [20]. 

Cases 4 [21], 5 [22], 6 [23], 7 [24], 8 [25], 12 [29], and 17 [33] from Table 2 and Figure 8 represent the intermediate 4q partial trisomy of the 4q23–q31 region. All these cases presented a severe phenotype characterized by: growth retardation (7/7), intellectual disability (7/7), high nasal bridge (7/7), epicanthic folds (6/7), low set malformed ears (6/7), micrognathia (5/7), thumb anomalies (5/7), and short philtrum (5/7). However, the genes located in this region seem to have a lesser impact on the development of the heart because only one of these patients presented a congenital heart defect. Lundin et al. (2002) presumed that the 4q27.4–q31 partial trisomy induced the most severe clinical effects, including growth retardation, intellectual disability, microcephaly, facial asymmetry, thumb anomalies, hearing impairment, epilepsy, and congenital heart defects [31].

Terminal 4q partial trisomies are represented by Cases 13 [30], 14 [30], 20 [36], 21 [37], 23 [38], 26 [39], 28 [41], 29 [42], 31 [44], 37 [49], 41 [53], 42 [54], 43 [55], 44 [56], 45 [57], 47 [59], 48 [59], and 49 [60] (Table 2, Figure 8). The majority of these encompassed the 4q31-q35(qter) region. The main phenotypic changes were represented by low set malformed ears (16/18), intellectual disability (17/18), high nasal bridge (13/18), growth retardation (14/18), microcephaly (11/18) epicanthic folds (7/18), and micrognathia (8/18). Thumb anomalies (6/18) and short philtrum (5/18) are rare, while cardiac heart defects were found in only 4 cases. Renal anomalies could also be considered less frequent, being found in 6/18 cases. 

The majority of the cases presented in Table 2 and Figure 8 are represented by large 4q partial trisomies that encompassed at least the 4q27–qter region (Cases 15 [31], 16 [32], 22 [6], 24 [7], 25 [9], 27 [40], 32 [45], 33 [46], 34 [47], 35 ([48], case A), 36 ([48], case B), 38 [50], 39 [51], 40 [52], 46 [58], and our case). The phenotype of these patients is severe, being correlated with the large amount of genetic material present in the three copies. The major anomalies reported are: growth retardation (15/16), low set malformed ears (13/16), intellectual disability (13/16), microcephaly (14/16), high nasal bridge (13/16), and micrognathia (12/16). Epicanthic folds (7/16) and short philtrum (6/16) are not very frequent. Visceral anomalies are relatively frequent, congenital heart disease having been documented in 7 cases and renal anomalies in 6 cases. 

Rinaldi et al. (2005) discussed the correlation between 4q partial trisomy and the phenotype and observed an important clinical heterogeneity correlated with the variability of the duplicated region. They considered that psychomotor and growth retardation, retromicrognathia, and low set and/or malformed ears are non-specific clinical findings associated with chromosomal unbalances [40].

The relationship between 4q partial trisomy and renal anomalies has been discussed by different authors but still lacks certitude. Zollino et al. (1995) suggested that the 4q22–q23 region is involved in the development of the acrorenal field [19]. On the other hand, Battaglia et al. (2005) considered that the development of the acrorenal field is more likely associated with genes from the 4q25–q28 region [8]. Otsuka et al. (2005) suggested that renal hypoplasia may have a close relationship with the duplication of 4q33–q34 [30]. This fact is also suggested by the elements presented in Table 2. Thus, all the cases with renal anomalies presented a minimum of a 4q33–qter partial trisomy. 

Congenital cardiac defects were present in only 14 of 50 patients with 4q partial trisomy (see Table 2). In seven of those cases, the presence of congenital heart disease could be considered a consequence of supplementary genetic material from chromosome 4 because they had either a 4q duplication [9,20,25,26,50] or a translocation, but the monosomy of the other chromosome did not alter the phenotype [35,40]. In the rest of the seven cases, an association between 4q partial trisomy and monosomy of chromosomes 13 [38], 18 [39,48], 20 [51], 21 [58], 5 [56], and 10 (our case) was present, and thus, the delineation of a chromosomal region involved in the development of congenital heart disease is difficult. However, Rinaldi et al. (2005) presumed that the 4q26-q27 region could be the critical region that contains genes for congenital development; this segment is duplicated in 9 of the 12 cases discussed above [40]. The *HAND2* gene (4q34.1) is implicated in cardiac morphogenesis. The effects on the phenotype are different for haploinsufficiency and for duplication of the *HAND2* gene because only haploinsufficiency seems to be associated with congenital heart defects [57]. 

Thumb anomalies are considered a particularity of 4q partial trisomy, but the data from Table 2 indicate such an anomaly in only 17 out of 50 cases. The analysis of these data enables us to presume that the 4q27–q28 segment could be the region that contains genes involved in thumb development because all cases (except for the siblings reported by Otsuka et al., 2005 and Lin, 2018, Thapa case B) presented 4q duplication that encompasses this segment [30,56,59]. A similar hypothesis formulated by Rinaldi et al. (2005) indicates that the 4q25-q28 region is a minimal commonly duplicated segment [40]. 

The 10q subtelomeric deletion is a rare anomaly. The symptomatology of this syndrome includes: variable dysmorphic facial features (microcephaly, dolichocephaly, triangular asymmetric face, prominent nasal root with beaked nose and flared nostrils, long philtrum, thin upper lip, small pointed jaw, malformed and low set ears, low posterior hairline), delayed psychomotor development, and poor speech and language development [11,14,63,64,65]. In all these cases, the chromosomal deletion was of minimum 5 Mb and encompassed a region located between the 10q26 band and the 10q telomere. Yatsenko et al. (2009) considered that the minimum critical region is approximately 600 kb, and it is located in the 10q26.2 region [15]. In this segment are present genes *DOCK1* and *C10ORF90*. The *DOCK1* gene influences phagocytosis, cell migration, apoptosis and tumorigenesis, and cardiovascular development. Lin et al. (2016) suggested that the distal 10q26 terminal deletion with a breakpoint at ~130.0 Mb from the 10p telomere may be involved in the phenotype of the 10q26 deletion syndrome, but this could be influenced by other pathogenic genes: *FGFR2*, *CTBP2*, *CALY, WDR11, HMX2,* and *HMX3* [16]. A similar hypothesis was proposed by Sangu et al. (2016), who presented a patient with a de novo microdeletion of 10q26.11-q26.13, where genes *FGFR2, HMX2,* and *HMX3* are located [66]. 

**Figure 9:**
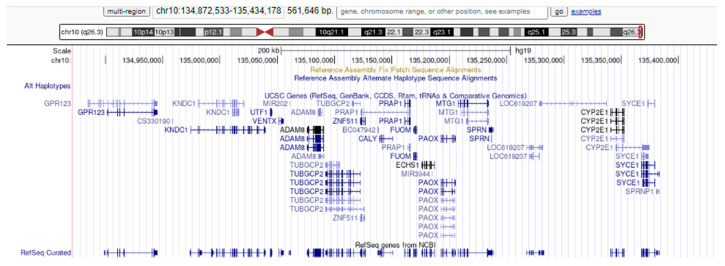
Genome Browser image of genes in the 10q26.3 region.

However, in our case, the 10q26.3 microdeletion is located much closer to the 10q telomere, and the dimension is only 562 kb. The 10q26.3 region contains 27 genes, 18 of which encode proteins (Figure 9). Three of these, the genes *ECHS1* (enoyl-CoA hydratase, short chain 1), *SYCE1* (synaptonemal complex central element protein 1), and *TUBGCP2* (tubulin gamma complex associated protein 2) have been associated with autosomal recessive disorders [67]. Therefore, the absence of a gene copy in our patient is not involved in phenotypic changes. This is concordant with other reports. Martin et al. (2002) presented two relatives (mother and child) that had a 10q telomere deletion with minimum phenotypic changes (mother healthy; child with some unspecified neurological features) [68]. A similar case was discussed by Ravnan et al. (2006), who identified a mother–child transmission of a 10q telomeric deletion and considered this anomaly to be a chromosomal variant [69]. Zhang et al. (2009), who presented an anomaly similar to that reported by us, considered that the 10q26.3 microdeletion was without phenotypic consequences and all phenotypic changes in their patient resulted from the 4q26–q35.2 partial trisomy [7]. Riegel et al. (2001) reported two siblings with a der(10)t(4;10)(q35.2;q26.3) anomaly that presented moderate intellectual disability, dysmorphic features (triangular face; hypertelorism; downward slanting palpebral fissures; short, upturned nose with broad and prominent root and hypoplastic alae; thin upper lip; downturned corners of the mouth; bilateral preauricular tags), smooth palmar creases, and short fingers [70]. Yan et al. (2014) presented a 10q26.3 microdeletion (arr10q26.3(131,585,685–134,832,720)x1) identified prenatally in a child with a ventricular septal defect, overriding aorta, and right ventricular hypertrophy [71]. The absent segment, in this case, does not encompass the 10q chromosomal region identified by us. Considering all this information, we presume that the 10q26.3 microdeletion had minimal influence on the phenotype of our patient. This is concordant with the theory postulated by Kowalczyk et al. (2007), who explained the absence of phenotypic changes in microdeletion by several facts: the lost material is non-essential; the genes located in the deleted region are haplosufficient; some deleted genes have additional copies, which can compensate for the loss; the chromosomal region is imprinted and the remaining allele on the other chromosome of the pair is active [72].

The fragment of 795 kb located in the 4q35.2 region, present in three copies in the mother and in four copies in the child, can be considered non-pathogenic, although it contains the genes *ZFP42, TRIML1,* and *TRIML2.* All these genes encode proteins that are members of the superfamily of zinc-finger. The *ZFP42* gene is not involved in human pathology, while mice homologous gene *Zfp42* intervenes in the reprogramming of X chromosome inactivation [73]. Genes *TRIML1* and *TRIML2* are involved in early embryogenesis [74]. 

Human duplication is favored by the existence of low copy repeat sequences involved in homologous recombination events mediated by high sequence identity between the low copy repeats [75]. Abnormal recombination induces chromosomal rearrangements involved in genomic disorders such as Williams-Beuren syndrome [76], Smith-Magenis syndrome and the corresponding 17p11.2 duplication [77], and the DiGeorge/velo-cardio-facial syndrome and cat-eye syndrome produced by rearrangements in the 22q11 region [78]. 

In other cases, such chromosomal changes do not modify the phenotype and generate only a copy number variation (CNV) [79]. Some duplications (or even triplication/quadruplication) without phenotype changes were identified on the long arm of chromosome 4. For example, Chen et al. (2014) reported a 4.5 Mb benign 4q12–q13.1 triplication transmitted from father to son, and Chen et al. (2012) presented a 4.4 Mb benign 4q12-q13.1 quadruplication transmitted from parent to child [80,81]. 

Bertelsen et al. (2014) reported a patient with Tourette syndrome that associated a 1.7 Mb benign duplication in the 4q35.2 region (chr4.hg19:g.(?_188104036)_(189795272_?)), transmitted from father to child [82]. The duplicated segment includes *ZFP42*, *TRIML1*, and *TRIML2* genes. This duplication is similar to the anomaly discovered by us, and, thus, we can conclude that the duplication in the 4q35.2 region is without phenotypic changes. The 4q35.2 duplication could be favored by the genomic architecture of this chromosomal region. Close to the region that contains the *ZFP42*, *TRIML1*, and *TRIML2* genes is located an array of 3.3 kb repeats (D4Z4). The contraction of these repeats from 11–100 copies to ≤10 generates facio-scapulo-humeral muscular dystrophy and can modify the organization of chromatin in this segment of chromosome 4. In addition, the D4Z4 repeats of chromosome 4 have a similar macrosatellite homologous segment in the 10q26.3 region [83].

Matsumura et al. (2002) described a high rate of translocation between the 4q35.2 region and the 10q26.3 region in both Asiatic and European populations, and these rearrangements are favored by genomic homology between these chromosomal segments [84]. Starting from this, we can presume that this homology could favor the chromosomal rearrangements between chromosomes 4 and 10, including the complex rearrangements described by us. 

In these conditions, prenatal diagnosis of the future pregnancies of the mother becomes mandatory due to the theoretical 2/3 risk of forming fetuses with unbalanced chromosomal abnormalities. However, the practical risk is only around 5% because viable unbalanced embryos can result from an adjacent-1 segregation of maternal derivative chromosomes and the embryos carrying the association between 4q trisomy and 10q monosomy. The embryos with an association between 10q trisomy and 4q monosomy (generated by an adiacent-1 segregation) and those that result from an adjacent-2 segregation of maternal derivative chromosomes are unviable [85,86,87,88].

## 5. Conclusions

We present a rare complex chromosomal anomaly identified in one patient with multiple congenital anomalies syndrome. The boy has a chromosomal formula of 46,XY,der(10)(10pter→10q26.2::4q26→4qter) and his mother presents an apparently balanced reciprocal translocation - 46,XX,t(4;10)(q26;q26.2). Using molecular analyses (MLPA and array-CGH), we confirmed the presence in our patient of an association between a large triplication of 71,057 kb in the 4q26–q35.2 region, a 562 kb microdeletion in the 10q26.3 region, and a 795 kb quadruplication of the 4q35.2 region. In these conditions, we consider that phenotype was modified mainly by the 4q partial trisomy, while the 10q26.3 microdeletion allowed a minimal influence on the phenotype; thus, we consider our case as a “pure” 4q partial trisomy. Because of the mother–son transmission of the 4q35.2 triplication, we consider that this genomic change is only a CNV without pathogenic importance. This report highlights the importance of the use of different cytogenetic and molecular methods to identify specific chromosomal anomalies present in some multiple congenital anomalies syndromes.

## Figures and Tables

**Table 1:** Different types of 4q partial trisomy.

No	Reference	Chromosome 4 Region	Type of Anomaly
1	Mattei et al., 1979 [18]	q12–q13	IH, t(2;4) mat
2	Zollino et al., 1995 [19]	q13.3–q22.2	DN, dup(4)(q13.3q22.2)
3	Halal et al., 1991 [20]	q23–q27	DN, dup(4)(q23q27)
4	Jeziorowska et al., 1993 [21]	q21.3–q31.3	DN, dup(4)(q21.3q31.3)
5	Fryns, 1980 [22]	q25–q31	DN, dup(4)(q25q31)
6	Vogel et al., 1975 [23]	q22–q34	DN, dup(4)(q22q34)
7	Dutrillaux et al., 1975 [24]	q22–q34	DN, dup(4)(q22q34)
8	Taylor et al., 1977 [25]	q26–q35	DN, dup(4)(q26q35)
9	Goodman et al., 1997 [26]	q31.1–q32.3	IH, dup(4)(q31.1q32.3)
10	Muraki et al., 1997 [27]	q25–q31.3	DN, dup(4)(q25q31.3)
11	Shashi et al., 1999 [28]	q12–q13	DN, dup(4)(q12q13)
12	Guillen Navarro et al., 1996 [29]	q21–q28	DN, dup(4)(q21q28)
13	Otsuka et al., Case 1, 2005 [30]	q31.22–q35.2	IH, dup(4)(q31.22q35.2)
14	Otsuka et al., Case 2, 2005 [30]	q31.22–q35.2	IH, dup(4)(q31.22q35.2)
15	Lundin et al., 2002 [31]	q27–q35	DN, t(4;7)(q27;p22)
16	Mikelsaar et al., 1996 [32]	q25–qter	DN, t(4;22)(q25;p11)
17	Cui et al., 2006 [33]	q27–q35	DN, t(4;5)(q27;q35)
18	Maltby et al., 1999 [34]	q31–q33	IH, dup(4)(q31q33)
19	Assawamakin et al., 2012 [35]	q13.2–q22.1	IH, der(8)(20qter–>20q12::4q22.1–>q21.21::4q13.3–>4q13.2::8q22.1–>8p11.12::8q22.3–>qter),der(20)(20pter –>20q12::4q13.3–>q21.21::8q22.3–>q22.1::8p11.12–>pter)
20	Angulo et al., 1984 [36]	q31–qter	IH, t(4;12)(q31;q24)
21	Watanabe et al., 1977 [37]	q33–qter	IH, t(4;13)(q33;q33)
22	Schrott et al., 1974 [6]	q26–qter	IH, t(4;13)(q26;q34)
23	Jenkins et al., 1975 [38]	q31–qter	DN, t(4;13)(q31;q14)
24	Zhang et al., 2009 [7]	q25–qter	IH, t(4;10)(q26;q26.3)
25	Cernakova et al., 2006 [9]	q28–q35.2	DN, dup(4)(q28q35.2)
26	Horbinski et al., 2008 [39]	q35–qter	DN, t(4;18)(q35;q23)
27	Rinaldi et al., 2005 [40]	q24–q35	DN, t(4;14)(q24;p12)
28	Kadotani et al., 1981 [41]	q32–qter	IH, t(4;9)(q23;p24)
29	Issa et al., 1976 [42]	q31–qter	IH, t(4;9)(q31;q34)
30	Collia et al., 2012 [43]	q12–q22	DN, dup(4)(q11q22)
31	Baccichetti et al., 1975 [44]	q32–qter	IH, t(4;21)(q32;q22)
32	Patel et al., 2006 [45]	q25–qter	IH, t(4;18)(q25;q22)
33	Cervenka et al., 1976 [46]	q25–qter	IH, t(X;4)(q27;q25)
34	Biederman and Bowen 1976 [47]	q21–qter	IH, t(2;4)(p25;q21)
35	Bonfante et al., case A, 1979 [48]	q27–qter	IH, t(4;18)(q27;p11)
36	Bonfante et al., case B, 1979 [48]	q27–qter	IH, t(4;18)(q27;p11)
37	Škrlec et al., 2014 [49]	q31.3–qter	IH, t(2;4)(p25.1;q31.3)
38	Gorukmez et al., 2014 [50]	q21–q35	DN, dup(4)(q21q35)
39	Vargas Machuca et al., 2016 [51]	q21–qter	IH, t(4;20)(q21;q13.1)
40	El-Ruby et al., 2007 [52]	q25–qter	IH, t(4;21)(q25;q22)
41	Anneren et al., 1984 [53]	q31–qter	IH, t(4;8)(q31;p23)
42	Carrascosa Romero et al., 2008 [54]	q31–q35	DN, dup(4)( q31q35)
43	Bellucco et al., 2018 [55]	q32.1–q35.2	47,XX,+der(21)t(4; 21)(q32.1;q21.2)mat. arr[GRCh37/hg19] 4q32. 1q35.2 (158907036_190957460)x3,21q11.2q21.2(15016486_25605895)x3
44	Lin et al., 2018 [56]	q32.3–qter	ND, t(4;5)(q32.3;p14.2)
45	Mohamed et al., 2018 [57]	q32.1–q35.2	DN 46,XX,add1(q44)
46	Shenoy et al., 2018 [58]	q27q35.2	IH, t(4;21)(q27;q22)
47	Thapa et al., case A, 2014 [59]	q32.1–q35.2	ND, dup(4)(q32.1q35.2)
48	Thapa et al., case B, 2014 [59]	q32.2–q34.3	ND,dup(4)(q32.2q34.3)
49	Zaki et al., 2019 [60]	q35.2	DN, dup(4)(q35.2)
50	This case	q26–qter	IH, t(4;10)(q26;q26.3)

IH—inherited; DN—de novo; ND—not discussed.

**Table 2:** Phenotype changes in different types of 4q partial trisomy.

No	Reference	Chromosome 4 Region	a	b	c	d	e	f	g	h	i	j	k
1	Mattei et al., 1979 [18]	q12–q13	+	+	+	+	−	+	−	−	−	−	−
2	Zollino et al., 1995 [19]	q13.3–q22.2	−	+	−	−	−	−	+	−	−	−	−
3	Halal et al., 1991 [20]	q23–q27	+	+	+	+	−	−	−	−	−	+	−
4	Jeziorowska et al., 1993 [21]	q21.3–q31.3	+	+	+	+	+	+	+	+	+	−	+
5	Fryns, 1980 [22]	q25–q31	+	+	−	−	+	+	−	+	−	−	−
6	Vogel et al., 1975 [23]	q22–q34	+	+	−	+	+	+	+	+	+	−	+
7	Dutrillaux et al., 1975 [24]	q22–q34	+	+	+	+	+	+	+	+	+	−	+
8	Taylor et al., 1977 [25]	q26–q35	+	+	+	+	+	+	+	+	−	+	ND
9	Goodman et al., 1997 [26]	q31.1–q32.3	+	+	−	+	−	ND	ND	−	−	+	−
10	Muraki et al., 1997 [27]	q25–q31.3	+	+	−	−	−	−	−	+	−	−	−
11	Shashi et al., 1999 [28]	q12–q13	+	+	+	+	+	−	−	+	−	−	−
12	Guillen Navarro et al., 1996 [29]	q21–q28	+	+	−	+	+	ND	+	+	+	−	−
13	Otsuka et al., Case 1, 2005 [30]	q31.22–q35.2	+	+	+	+	+	+	+	+	+	−	+
14	Otsuka et al., Case 2, 2005 [30]	q31.22–q35.2	+	+	+	+	+	+	+	+	+	−	+
15	Lundin et al., 2002 [31]	q27–q35	+	+	+	+	+	ND	−	−	+	−	−
16	Mikelsaar et al., 1996 [32]	q25–qter	+	+	+	+	+	+	−	+	−	−	−
17	Cui et al., 2006 [33]	q27–q35	+	+	−	+	+	ND	−	−	+	−	−
18	Maltby et al., 1999 [34]	q31–q33	−	−	−	−	−	ND	−	−	−	−	−
19	Assawamakin et al., 2012 [35]	q13.2–q22.1	−	+	+	−	−	ND	−	−	−	+	−
20	Angulo et al., 1984 [36]	q31–qter	+	+	−	+	+	+	ND	+	−	−	+
21	Watanabe et al., 1977 [37]	q33–qter	+	+	+	−	+	ND	−	+	ND	−	+
22	Schrott et al., 1974 [6]	q26–qter	+	+	+	−	−	ND	+	−	ND	−	+
23	Jenkins et al., 1975 [38]	q31–qter	+	+	−	+	+	ND	−	+	ND	+	−
24	Zhang et al., 2009 [7]	q25–qter	+	+	+	+	+	+	+	+	−	−	−
25	Cernakova et al., 2006 [9]	q28–q35.2	+	+	+	+	+	−	+	+	−	+	−
26	Horbinski et al., 2008 [39]	q35–qter	+	+	+	+	+	−	−	+	−	+	−
27	Rinaldi et al., 2005 [40]	q24–q35	+	+	+	ND	+	ND	+	+	+	+	+
28	Kadotani et al., 1981 [41]	q32–qter	+	ND	+	ND	+	ND	+	+	+	ND	ND
29	Issa et al., 1976 [42]	q31–qter	+	+	−	ND	+	ND	−	+	−	−	ND
30	Collia et al., 2012 [43]	q12–q22	−	−	−	+	+	ND	−	+	−	−	−
31	Baccichetti et al., 1975 [44]	q32–qter	+	+	+	−	+	−	+	+	−	ND	−
32	Patel et al., 2006 [45]	q25–qter	+	+	+	ND	+	ND	+	+	+	−	+
33	Cervenka et al., 1976 [46]	q25–qter	+	+	+	+	+	−	−	+	+	−	+
34	Biederman and Bowen, 1976 [47]	q21–qter	+	+	+	ND	+	+	+	+	+	−	ND
35	Bonfante et al., case A, 1979 [48]	q27–qter	+	+	−	−	−	−	+	+	−	+	−
36	Bonfante et al., case B, 1979 [48]	q27–qter	+	+	−	+	+	−	−	+	−	−	−
37	Škrlec et al., 2014 [49]	q31.3–qter	−	+	−	+	−	ND	ND	+	−	−	−
38	Gorukmez et al., 2014 [50]	q21–q35	+	+	+	−	+	+	+	+	+	+	+
39	Vargas Machuca et al., 2016 [51]	q21–qter	+	ND	+	ND	+	ND	+	+	−	+	−
40	El-Ruby et al., 2007 [52]	q25–qter	+	+	+	−	+	−	−	+	+	−	−
41	Anneren et al., 1984 [53]	q31–qter	+	+	+	+	+	ND	−	+	−	−	−
42	Carrascosa Romero et al., 2008 [54]	q31–q35	−	+	+	ND	+	+	+	+	−	−	+
43	Bellucco et al., 2018 [55]	q32.1–q35.2	+	+	+	−	+	−	−	−	−	−	−
44	Lin et al., 2018 [56]	q32.3–qter	ND	+	ND	ND	ND	ND	ND	ND	+	+	+
45	Mohamed et al., 2018 [57]	q32.1–q35.2	+	+	+	−	−	ND	+	+	−	−	−
46	Shenoy et al., 2018 [58]	q27q35.2	+	ND	+	ND	ND	ND	+	+	ND	+	+
47	Thapa et al., case A, 2014 [59]	q32.1–q35.2	+	+	ND	−	+	+	+	+	−	ND	−
48	Thapa et al., case B, 2014 [59]	q32.2–q34.3	+	+	ND	−	ND	ND	−	+	+	ND	−
49	Zaki et al., 2019 [60]	q35.2	−	+	+	ND	ND	ND	ND	+	−	−	−
50	This case	q26–qter	+	+	+	+	+	+	+	+	−	+	−

ND—not discussed; a—growth retardation; b—psychomotor retardation; c—microcephaly; d—epicanthic folds; e—high nasal bridge; f—short philtrum; g—micrognathia; h—low set and malformed ears; i—thumb anomalies; j—congenital heart diseases; k—renal anomalies.
